# (1′*S*)-4-(3,4-Dichlorophenyl)-1′-(3,5-dimethoxyphenyl)-1,2,3,4-tetrahydronaphthalene-2-spiro-2′-pyrrolizidine-3′-spiro-3′′-indoline-1,2′′-dione

**DOI:** 10.1107/S1600536808028614

**Published:** 2008-09-17

**Authors:** E. Theboral Sugi Kamala, R. Murugan, S. Nirmala, L. Sudha, S. Sriman Narayanan

**Affiliations:** a Department of Physics, Easwari Engineering College, Ramapuram, Chennai 600 089, India; b Department of Analytical Chemistry, University of Madras, Guindy Campus, Chennai 600 025, India.; c Department of Physics, SRM University, Ramapuram Campus, Chennai 600 089, India

## Abstract

In the title compound C_37_H_32_Cl_2_N_2_O_4_, the unsubstituted pyrrolidine ring shows a twist conformation whereas the substituted pyrrolidine ring shows an envelope conformation. The dimeth­oxy benzene ring is perpendicular to the tetra­lone ring, making a dihedral angle of 89.94 (5)°. Mol­ecules are linked into centrosymmetric dimers by N—H⋯O hydrogen bonds and the crystal structure is stabilized by C—H⋯π inter­actions and C—H⋯O hydrogen bonds. One meth­oxy group is disordered over two positions with the site occupancy factors of 0.84 (2) and 0.16 (2).

## Related literature

For general background, see: Ma & Hecht (2004 ▶); Mitsuaki *et al.* (1997 ▶); Raghunathan  & Suresh Babu (2006 ▶); Reddy & Rao (2006 ▶); Usui *et al.* (1998 ▶). For bond-length data, see: Allen *et al.* (1987 ▶). For puckering parameters, see: Cremer & Pople (1975 ▶). For asymmetry parameters, see: Nardelli (1983 ▶).
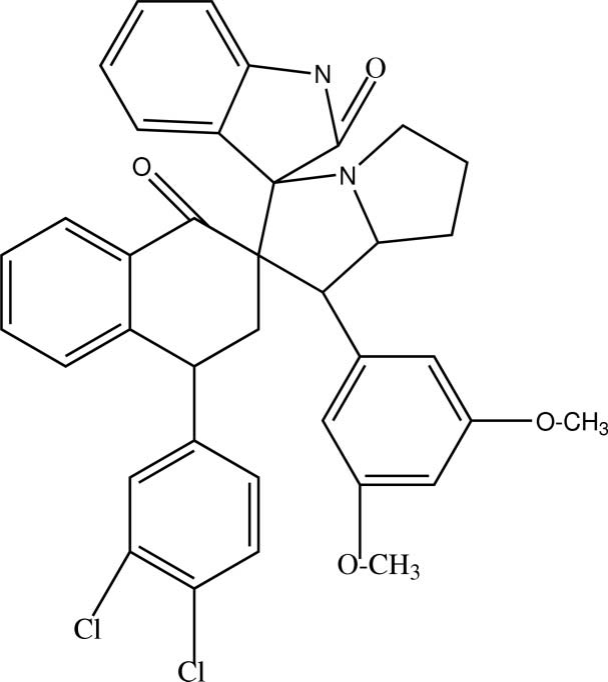


## Experimental

### 

#### Crystal data

C_37_H_32_Cl_2_N_2_O_4_
*M* *_r_* = 639.55
Triclinic, 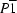

*a* = 10.4475 (3) Å
*b* = 11.3047 (3) Å
*c* = 15.0170 (4) Å
α = 87.925 (2)°
β = 70.3220 (10)°
γ = 70.115 (2)°
*V* = 1564.29 (7) Å^3^
*Z* = 2
Mo *K*α radiation
μ = 0.25 mm^−1^
*T* = 293 (2) K
0.40 × 0.20 × 0.20 mm

#### Data collection

Bruker Kappa APEXII diffractometer
Absorption correction: multi-scan (Blessing, 1995 ▶) *T*_min_ = 0.852, *T*_max_ = 0.943
41544 measured reflections
10800 independent reflections
7229 reflections with *I* > 2σ(*I*)
*R*_int_ = 0.026

#### Refinement

*R*[*F* ^2^ > 2σ(*F* ^2^)] = 0.056
*wR*(*F* ^2^) = 0.191
*S* = 1.04
10800 reflections
414 parameters
3 restraints
H-atom parameters constrained
Δρ_max_ = 0.79 e Å^−3^
Δρ_min_ = −0.44 e Å^−3^

### 

Data collection: *APEX2* (Bruker, 2004 ▶); cell refinement: *APEX2* and *SAINT* (Bruker, 2004 ▶); data reduction: *SAINT* and *XPREP* (Bruker, 2004 ▶); program(s) used to solve structure: *SHELXS86* (Sheldrick, 2008 ▶); program(s) used to refine structure: *SHELXL97* (Sheldrick, 2008 ▶); molecular graphics: *ORTEP* (Farrugia, 1997 ▶); software used to prepare material for publication: *SHELXL97*.

## Supplementary Material

Crystal structure: contains datablocks I, global. DOI: 10.1107/S1600536808028614/bt2783sup1.cif

Structure factors: contains datablocks I. DOI: 10.1107/S1600536808028614/bt2783Isup2.hkl

Additional supplementary materials:  crystallographic information; 3D view; checkCIF report

## Figures and Tables

**Table 1:** Hydrogen-bond geometry (Å, °)

*D*—H⋯*A*	*D*—H	H⋯*A*	*D*⋯*A*	*D*—H⋯*A*
N2—H2⋯O1^i^	0.86	2.01	2.8566 (16)	166
C37—H37*C*⋯*Cg*1^ii^	0.93	2.75	3.493 (2)	134
C4—H4⋯O1	0.98	2.56	3.0989 (16)	114
C5—H5⋯O2	0.98	2.27	2.790 (2)	112
C13—H13⋯O2	0.93	2.57	3.129 (2)	119
C23—H23*A*⋯O1	0.97	2.35	3.015 (2)	125
C22—H22⋯N2	0.98	2.55	3.447 (2)	152

Symmetry codes: (i) 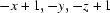; (ii) 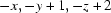. *Cg*1 is the centroid of the C30–C35 ring.

**Fig. 1.**
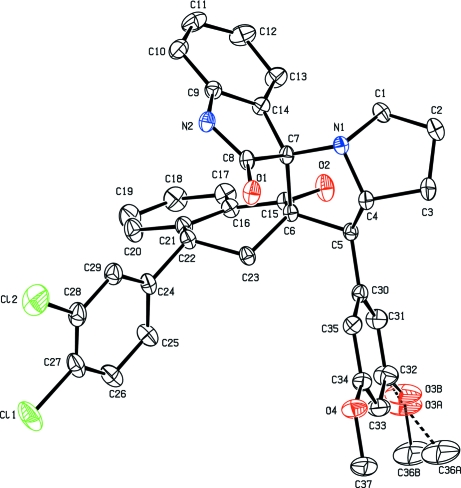
The molecular structure of the title compound with 30% probability displacement ellipsoids.

**Fig. 2.**
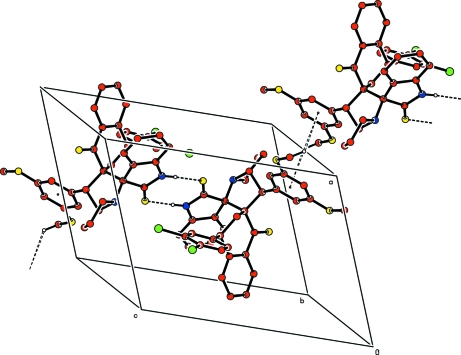
The packing of the molecules viewed along the c axis.

## supplementary crystallographic information

### Comment

Spiro compounds represent an important class of naturally occurring substances exhibiting significant biological properties. The spirooxindole system is the core structure of many pharmacological agents and natural alkaloids (Ma & Hecht, 2004). Spirotryprostatin A, a natural alkaloid isolated from the fermentation broth of Aspergillus fumigatus, has been identified as a novel inhibitor of microtubule assembly (Usui *et al.*, 1998). Because of their synthetic and biological potential, considerable interest has been focused on the synthesis of spirooxindole derivatives *via* 1,3-dipolar cycloaddition reactions (Raghunathan & Suresh Babu, 2006). Chiral polyhydroxy alkaloids show remarkable biological properties. Among these, pyrrolidine alkaloids carrying an aromatic substituent on the ring are of a rare class found in nature (Reddy & Rao, 2006).They are useful in preventing and treating rheumatoid arthritis, asthma, allergies, rhinitis and related diseases (Mitsuaki *et al.*, 1997).

Fig 1 shows the *ORTEP* (Farrugia,  1997) plot of the title compound. Bond lengths and angles are comparable with other reported values (Allen *et al.*,1987).

In the molecule the pyrrolidine ring N1/C4—C7 and the tetralone ring C6/C15/C16/C21/C22/C23 exhibit *envelope* conformations with the asymmetry parameters (Nardelli, 1983) ΔC_s_(C4)/C(16) = 5.20 (14)/2.64 (15) and with the puckering parameters (Cremer & Pople, 1975) q2 = 0.3779 (16) Å and 0.4787 (17)Å and φ2 =212.1 (2)° / 124.3 (3)°. The pyrrolidine ring N1/C1—C4 exhibits a *twist* conformation with assymetry parameter ΔC_s_ (N1) =16.01 (18), ΔC_2_ (C1) =62.94 (18) and with the puckering parameters q2 = 0.3901 (18) Å, φ2 = 18.9 (3)°.

The sum of bond angles around N1 [329.84°] and that around atom N2 [360.00°] indicate *sp*^3^ and *sp*^2^ hybridizations. The dimethoxy benzene ring C30—C35 is perpendicular to the tetralone ring C6/C15/C16/C21/C22/C23 and the phenyl ring C16—C21 making a dihedral angle of 89.94 (5)° and 89.98 (6)° respectively. The phenyl and the tetralone rings are almost coplanar with each other making a dihedral angle of 6.53 (5)°.

In the crystal packing, atoms O1 and N2 are involved in intermolecular N—H···O interactions and atoms O1 and O2 are involved in intramolecular C-H···O interactions. The molecules pack into distinct layers facilitated by C-H···π interactions.

### Experimental

1.0 mol of (2E)-4-(3,4-dichlorophenyl)-2-(3,5-dimethoxybenzylidene)-3, 4-dihydronaphthalen-1(2*H*)-one (1.0 g), 1.0 mol of isatin (0.33 g) and 1.0 mol of *L*-proline were refluxed in methanol at 65°C for about 5.0 hrs with constant stirring to afford the cycloadduct. The reaction mixture was monitored by TLC. After the completion of reaction, the reaction mixture was allowed to cool. The solvent was removed by vacuum, the crude solid was purified by column chromatography using n-hexane: ethylacetate (8:2). The cycloadduct was recrystallized by chloroform: methanol (9:1).

### Refinement

Atoms C36 and O3 are disordered over two positions (C36B/C36A) and (O3B/O3A) with refined occupancies of 0.840 (16) and 0.160 (16). H atoms were placed in idealized positions and allowed to ride on their parent atoms, with C–H = 0.93 or 0.96Å and *U*_iso_(H)= 1.2–1.5*U*_eq_(C).

### Crystal data

**Table:**

C_37_H_32_Cl_2_N_2_O_4_	*Z* = 2
*M**_r_* = 639.55	*F*(000) = 668
Triclinic, *P*1	*D*_x_ = 1.358 Mg m^−^^3^
Hall symbol: -P 1	Mo *K*α radiation, λ = 0.71073 Å
*a* = 10.4475 (3) Å	Cell parameters from 7586 reflections
*b* = 11.3047 (3) Å	θ = 2.9–30.0°
*c* = 15.0170 (4) Å	µ = 0.25 mm^−^^1^
α = 87.925 (2)°	*T* = 293 K
β = 70.322 (1)°	Prismatic, colourless
γ = 70.115 (2)°	0.40 × 0.20 × 0.20 mm
*V* = 1564.29 (7) Å^3^	

### Data collection

**Table:**

Bruker Kappa APEXII diffractometer	10800 independent reflections
Radiation source: fine-focus sealed tube	7229 reflections with *I* > 2σ(*I*)
graphite	*R*_int_ = 0.026
ω and φ scan	θ_max_ = 32.0°, θ_min_ = 1.5°
Absorption correction: multi-scan (Blessing, 1995)	*h* = −15→15
*T*_min_ = 0.852, *T*_max_ = 0.943	*k* = −16→16
41544 measured reflections	*l* = −22→22

### Refinement

**Table:**

Refinement on *F*^2^	Primary atom site location: structure-invariant direct methods
Least-squares matrix: full	Secondary atom site location: difference Fourier map
*R*[*F*^2^ > 2σ(*F*^2^)] = 0.056	Hydrogen site location: inferred from neighbouring sites
*wR*(*F*^2^) = 0.191	H-atom parameters constrained
*S* = 1.04	*w* = 1/[σ^2^(*F*_o_^2^) + (0.1047*P*)^2^ + 0.2966*P*] where *P* = (*F*_o_^2^ + 2*F*_c_^2^)/3
10800 reflections	(Δ/σ)_max_ = 0.002
414 parameters	Δρ_max_ = 0.79 e Å^−^^3^
3 restraints	Δρ_min_ = −0.44 e Å^−^^3^

### Special details

**Table:**

Geometry. All e.s.d.'s (except the e.s.d. in the dihedral angle between two l.s. planes) are estimated using the full covariance matrix. The cell e.s.d.'s are taken into account individually in the estimation of e.s.d.'s in distances, angles and torsion angles; correlations between e.s.d.'s in cell parameters are only used when they are defined by crystal symmetry. An approximate (isotropic) treatment of cell e.s.d.'s is used for estimating e.s.d.'s involving l.s. planes.
Refinement. Refinement of *F*^2^ against ALL reflections. The weighted *R*-factor *wR* and goodness of fit *S* are based on *F*^2^, conventional *R*-factors *R* are based on *F*, with *F* set to zero for negative *F*^2^. The threshold expression of *F*^2^ > σ(*F*^2^) is used only for calculating *R*-factors(gt) *etc*. and is not relevant to the choice of reflections for refinement. *R*-factors based on *F*^2^ are statistically about twice as large as those based on *F*, and *R*- factors based on ALL data will be even larger.

### Fractional atomic coordinates and isotropic or equivalent isotropic displacement parameters (Å^2^)

**Table:**

	*x*	*y*	*z*	*U*_iso_*/*U*_eq_	Occ. (<1)
O3A	0.354 (4)	0.472 (3)	1.074 (3)	0.0809 (9)	0.161 (16)
C36A	0.221 (4)	0.580 (2)	1.132 (2)	0.096 (2)	0.161 (16)
H36A	0.2439	0.6186	1.1779	0.145*	0.161 (16)
H36B	0.1444	0.5491	1.1640	0.145*	0.161 (16)
H36C	0.1911	0.6419	1.0906	0.145*	0.161 (16)
O3B	0.3543 (6)	0.4485 (4)	1.0667 (4)	0.0809 (9)	0.839 (16)
C36B	0.3052 (11)	0.5792 (4)	1.1031 (5)	0.096 (2)	0.839 (16)
H36D	0.3337	0.5836	1.1570	0.145*	0.839 (16)
H36E	0.2015	0.6148	1.1218	0.145*	0.839 (16)
H36F	0.3480	0.6258	1.0546	0.145*	0.839 (16)
C1	0.40416 (19)	−0.15566 (17)	0.86677 (11)	0.0399 (4)	
H1A	0.4676	−0.1445	0.8978	0.048*	
H1B	0.4391	−0.2431	0.8417	0.048*	
C2	0.2476 (2)	−0.1147 (2)	0.93408 (14)	0.0529 (5)	
H2A	0.1945	−0.1581	0.9145	0.063*	
H2B	0.2437	−0.1332	0.9983	0.063*	
C3	0.18444 (18)	0.02583 (18)	0.92877 (11)	0.0403 (4)	
H3A	0.1728	0.0720	0.9859	0.048*	
H3B	0.0908	0.0483	0.9211	0.048*	
C4	0.29422 (15)	0.05502 (14)	0.84187 (9)	0.0283 (3)	
H4	0.2457	0.1077	0.8012	0.034*	
C5	0.39284 (15)	0.11276 (14)	0.86608 (9)	0.0263 (3)	
H5	0.4163	0.0694	0.9193	0.032*	
C6	0.53615 (14)	0.07076 (14)	0.77879 (9)	0.0250 (3)	
C7	0.52504 (14)	−0.04945 (14)	0.73003 (9)	0.0262 (3)	
C8	0.49813 (15)	−0.01974 (15)	0.63454 (9)	0.0282 (3)	
C9	0.70601 (16)	−0.19044 (15)	0.60014 (10)	0.0308 (3)	
C10	0.83223 (19)	−0.28541 (17)	0.54864 (12)	0.0421 (4)	
H10	0.8617	−0.2977	0.4829	0.051*	
C11	0.9135 (2)	−0.36187 (19)	0.59893 (15)	0.0507 (5)	
H11	1.0000	−0.4261	0.5663	0.061*	
C12	0.8683 (2)	−0.34426 (19)	0.69656 (15)	0.0518 (5)	
H12	0.9223	−0.3988	0.7291	0.062*	
C13	0.74268 (19)	−0.24584 (17)	0.74662 (12)	0.0418 (4)	
H13	0.7136	−0.2334	0.8123	0.050*	
C14	0.66109 (16)	−0.16654 (15)	0.69812 (10)	0.0303 (3)	
C15	0.66684 (15)	0.02854 (14)	0.81252 (10)	0.0286 (3)	
C16	0.80579 (15)	0.04095 (15)	0.74984 (10)	0.0312 (3)	
C17	0.92621 (18)	−0.00570 (18)	0.77891 (13)	0.0403 (4)	
H17	0.9172	−0.0422	0.8362	0.048*	
C18	1.05792 (19)	0.0018 (2)	0.72365 (15)	0.0496 (5)	
H18	1.1374	−0.0287	0.7437	0.059*	
C19	1.07096 (19)	0.0547 (2)	0.63859 (16)	0.0528 (5)	
H19	1.1597	0.0602	0.6013	0.063*	
C20	0.95378 (18)	0.09990 (19)	0.60787 (13)	0.0453 (4)	
H20	0.9648	0.1347	0.5499	0.054*	
C21	0.81919 (16)	0.09383 (15)	0.66302 (11)	0.0331 (3)	
C22	0.69137 (16)	0.14085 (15)	0.62944 (10)	0.0310 (3)	
H22	0.6950	0.0710	0.5908	0.037*	
C23	0.54951 (15)	0.17705 (14)	0.71400 (10)	0.0290 (3)	
H23A	0.4691	0.2027	0.6905	0.035*	
H23B	0.5415	0.2492	0.7513	0.035*	
C24	0.69205 (16)	0.25062 (16)	0.56849 (10)	0.0340 (3)	
C25	0.6990 (2)	0.35991 (18)	0.60064 (13)	0.0441 (4)	
H25	0.7052	0.3663	0.6605	0.053*	
C26	0.6968 (2)	0.4603 (2)	0.54514 (15)	0.0501 (4)	
H26	0.7006	0.5339	0.5681	0.060*	
C27	0.68903 (19)	0.45170 (19)	0.45629 (14)	0.0484 (5)	
C28	0.6784 (2)	0.3440 (2)	0.42390 (12)	0.0469 (4)	
C29	0.68005 (19)	0.24370 (18)	0.47998 (11)	0.0408 (4)	
H29	0.6731	0.1711	0.4579	0.049*	
C30	0.32734 (16)	0.25188 (15)	0.89804 (9)	0.0297 (3)	
C31	0.3666 (2)	0.29472 (17)	0.96688 (12)	0.0407 (4)	
H31	0.4317	0.2381	0.9915	0.049*	
C32	0.3092 (2)	0.42142 (19)	0.99891 (13)	0.0502 (5)	
C33	0.2127 (2)	0.50766 (17)	0.96333 (13)	0.0473 (4)	
H33	0.1751	0.5928	0.9847	0.057*	
C34	0.17306 (18)	0.46448 (16)	0.89500 (12)	0.0379 (3)	
C35	0.23008 (17)	0.33770 (15)	0.86215 (11)	0.0344 (3)	
H35	0.2030	0.3102	0.8160	0.041*	
C37	0.0151 (2)	0.67044 (19)	0.88737 (17)	0.0568 (5)	
H37A	−0.0505	0.7131	0.8549	0.085*	
H37B	0.0905	0.7056	0.8740	0.085*	
H37C	−0.0369	0.6809	0.9545	0.085*	
Cl1	0.69362 (7)	0.57669 (6)	0.38722 (5)	0.0775 (2)	
Cl2	0.66031 (9)	0.33098 (7)	0.31522 (4)	0.0859 (2)	
N1	0.39275 (13)	−0.06905 (12)	0.79190 (8)	0.0280 (2)	
N2	0.60572 (14)	−0.10446 (13)	0.56524 (8)	0.0320 (3)	
H2	0.6120	−0.1055	0.5067	0.038*	
O1	0.39534 (11)	0.06372 (12)	0.62338 (7)	0.0375 (3)	
O2	0.65885 (13)	−0.01379 (14)	0.88883 (8)	0.0455 (3)	
O4	0.07684 (15)	0.54052 (12)	0.85601 (10)	0.0506 (3)	

### Atomic displacement parameters (Å^2^)

**Table:**

	*U*^11^	*U*^22^	*U*^33^	*U*^12^	*U*^13^	*U*^23^
O3A	0.1439 (18)	0.046 (2)	0.0818 (16)	−0.0274 (16)	−0.0796 (14)	−0.0059 (13)
C36A	0.145 (6)	0.0662 (19)	0.098 (3)	−0.029 (3)	−0.071 (4)	−0.022 (2)
O3B	0.1439 (18)	0.046 (2)	0.0818 (16)	−0.0274 (16)	−0.0796 (14)	−0.0059 (13)
C36B	0.145 (6)	0.0662 (19)	0.098 (3)	−0.029 (3)	−0.071 (4)	−0.022 (2)
C1	0.0474 (9)	0.0381 (9)	0.0344 (7)	−0.0177 (7)	−0.0126 (6)	0.0138 (6)
C2	0.0580 (11)	0.0504 (11)	0.0450 (9)	−0.0294 (9)	−0.0016 (8)	0.0124 (8)
C3	0.0336 (8)	0.0522 (10)	0.0323 (7)	−0.0200 (7)	−0.0030 (6)	0.0059 (7)
C4	0.0247 (6)	0.0350 (8)	0.0252 (6)	−0.0112 (6)	−0.0081 (5)	0.0060 (5)
C5	0.0262 (6)	0.0302 (7)	0.0219 (5)	−0.0089 (5)	−0.0089 (5)	0.0060 (5)
C6	0.0228 (6)	0.0313 (7)	0.0225 (5)	−0.0093 (5)	−0.0105 (4)	0.0074 (5)
C7	0.0247 (6)	0.0335 (7)	0.0205 (5)	−0.0086 (5)	−0.0099 (4)	0.0063 (5)
C8	0.0268 (6)	0.0402 (8)	0.0218 (5)	−0.0144 (6)	−0.0112 (5)	0.0077 (5)
C9	0.0296 (7)	0.0339 (8)	0.0304 (6)	−0.0135 (6)	−0.0098 (5)	0.0024 (5)
C10	0.0374 (8)	0.0419 (9)	0.0388 (8)	−0.0106 (7)	−0.0052 (6)	−0.0078 (7)
C11	0.0344 (8)	0.0421 (10)	0.0636 (12)	−0.0005 (7)	−0.0134 (8)	−0.0135 (9)
C12	0.0436 (10)	0.0430 (11)	0.0640 (12)	0.0009 (8)	−0.0282 (9)	−0.0008 (9)
C13	0.0405 (8)	0.0404 (9)	0.0402 (8)	−0.0016 (7)	−0.0213 (7)	0.0033 (7)
C14	0.0289 (7)	0.0317 (7)	0.0292 (6)	−0.0074 (6)	−0.0119 (5)	0.0031 (5)
C15	0.0268 (6)	0.0343 (8)	0.0277 (6)	−0.0102 (6)	−0.0139 (5)	0.0066 (5)
C16	0.0260 (6)	0.0355 (8)	0.0350 (7)	−0.0112 (6)	−0.0139 (5)	0.0066 (6)
C17	0.0332 (8)	0.0479 (10)	0.0461 (9)	−0.0132 (7)	−0.0230 (7)	0.0101 (7)
C18	0.0274 (8)	0.0597 (12)	0.0649 (12)	−0.0129 (8)	−0.0227 (8)	0.0081 (9)
C19	0.0263 (8)	0.0627 (13)	0.0673 (12)	−0.0177 (8)	−0.0120 (8)	0.0120 (10)
C20	0.0312 (8)	0.0552 (11)	0.0483 (9)	−0.0182 (8)	−0.0102 (7)	0.0146 (8)
C21	0.0270 (7)	0.0362 (8)	0.0361 (7)	−0.0118 (6)	−0.0106 (5)	0.0070 (6)
C22	0.0289 (7)	0.0356 (8)	0.0300 (6)	−0.0134 (6)	−0.0107 (5)	0.0106 (5)
C23	0.0267 (6)	0.0334 (7)	0.0291 (6)	−0.0115 (6)	−0.0120 (5)	0.0115 (5)
C24	0.0289 (7)	0.0418 (9)	0.0321 (7)	−0.0147 (6)	−0.0103 (5)	0.0141 (6)
C25	0.0506 (10)	0.0470 (10)	0.0461 (9)	−0.0255 (8)	−0.0233 (8)	0.0173 (7)
C26	0.0482 (10)	0.0470 (11)	0.0628 (12)	−0.0253 (9)	−0.0214 (9)	0.0213 (9)
C27	0.0332 (8)	0.0518 (11)	0.0529 (10)	−0.0125 (8)	−0.0106 (7)	0.0285 (8)
C28	0.0403 (9)	0.0544 (11)	0.0338 (8)	−0.0059 (8)	−0.0097 (6)	0.0153 (7)
C29	0.0397 (8)	0.0461 (10)	0.0324 (7)	−0.0116 (7)	−0.0111 (6)	0.0088 (6)
C30	0.0292 (7)	0.0330 (8)	0.0251 (6)	−0.0094 (6)	−0.0088 (5)	0.0042 (5)
C31	0.0515 (10)	0.0395 (9)	0.0372 (8)	−0.0144 (8)	−0.0241 (7)	0.0052 (6)
C32	0.0727 (13)	0.0442 (10)	0.0438 (9)	−0.0215 (10)	−0.0311 (9)	0.0014 (7)
C33	0.0615 (11)	0.0319 (9)	0.0459 (9)	−0.0118 (8)	−0.0196 (8)	0.0000 (7)
C34	0.0379 (8)	0.0332 (8)	0.0382 (8)	−0.0084 (7)	−0.0118 (6)	0.0045 (6)
C35	0.0347 (7)	0.0347 (8)	0.0323 (7)	−0.0077 (6)	−0.0142 (6)	0.0027 (6)
C37	0.0566 (12)	0.0332 (10)	0.0729 (14)	−0.0035 (9)	−0.0251 (10)	0.0046 (9)
Cl1	0.0666 (4)	0.0733 (4)	0.0887 (4)	−0.0243 (3)	−0.0271 (3)	0.0574 (3)
Cl2	0.1112 (6)	0.0922 (5)	0.0393 (3)	−0.0105 (4)	−0.0339 (3)	0.0171 (3)
N1	0.0281 (6)	0.0322 (6)	0.0245 (5)	−0.0118 (5)	−0.0091 (4)	0.0064 (4)
N2	0.0337 (6)	0.0423 (7)	0.0208 (5)	−0.0128 (6)	−0.0108 (4)	0.0031 (5)
O1	0.0294 (5)	0.0553 (7)	0.0268 (5)	−0.0088 (5)	−0.0151 (4)	0.0099 (5)
O2	0.0385 (6)	0.0698 (9)	0.0344 (6)	−0.0197 (6)	−0.0209 (5)	0.0220 (5)
O4	0.0527 (8)	0.0337 (7)	0.0609 (8)	−0.0021 (6)	−0.0270 (6)	0.0026 (6)

### Geometric parameters (Å, °)

**Table:**

O3A—C36A	1.507 (10)	C15—O2	1.2134 (17)
O3A—C32	1.56 (3)	C15—C16	1.491 (2)
C36A—H36A	0.9600	C16—C17	1.398 (2)
C36A—H36B	0.9600	C16—C21	1.398 (2)
C36A—H36C	0.9600	C17—C18	1.376 (2)
O3B—C32	1.341 (5)	C17—H17	0.9300
O3B—C36B	1.449 (4)	C18—C19	1.375 (3)
C36B—H36D	0.9600	C18—H18	0.9300
C36B—H36E	0.9600	C19—C20	1.382 (3)
C36B—H36F	0.9600	C19—H19	0.9300
C1—N1	1.4724 (19)	C20—C21	1.395 (2)
C1—C2	1.522 (3)	C20—H20	0.9300
C1—H1A	0.9700	C21—C22	1.507 (2)
C1—H1B	0.9700	C22—C24	1.516 (2)
C2—C3	1.510 (3)	C22—C23	1.5278 (19)
C2—H2A	0.9700	C22—H22	0.9800
C2—H2B	0.9700	C23—H23A	0.9700
C3—C4	1.5315 (19)	C23—H23B	0.9700
C3—H3A	0.9700	C24—C25	1.376 (3)
C3—H3B	0.9700	C24—C29	1.384 (2)
C4—N1	1.4710 (19)	C25—C26	1.382 (3)
C4—C5	1.532 (2)	C25—H25	0.9300
C4—H4	0.9800	C26—C27	1.373 (3)
C5—C30	1.508 (2)	C26—H26	0.9300
C5—C6	1.5573 (18)	C27—C28	1.379 (3)
C5—H5	0.9800	C27—Cl1	1.7265 (18)
C6—C23	1.5323 (19)	C28—C29	1.387 (2)
C6—C15	1.5342 (19)	C28—Cl2	1.722 (2)
C6—C7	1.623 (2)	C29—H29	0.9300
C7—N1	1.4667 (17)	C30—C35	1.387 (2)
C7—C14	1.520 (2)	C30—C31	1.389 (2)
C7—C8	1.5558 (18)	C31—C32	1.383 (3)
C8—O1	1.2215 (18)	C31—H31	0.9300
C8—N2	1.3475 (19)	C32—C33	1.381 (3)
C9—C10	1.378 (2)	C33—C34	1.387 (3)
C9—C14	1.391 (2)	C33—H33	0.9300
C9—N2	1.399 (2)	C34—O4	1.365 (2)
C10—C11	1.387 (3)	C34—C35	1.387 (2)
C10—H10	0.9300	C35—H35	0.9300
C11—C12	1.379 (3)	C37—O4	1.413 (2)
C11—H11	0.9300	C37—H37A	0.9600
C12—C13	1.388 (3)	C37—H37B	0.9600
C12—H12	0.9300	C37—H37C	0.9600
C13—C14	1.383 (2)	N2—H2	0.8600
C13—H13	0.9300		
			
C36A—O3A—C32	105.7 (19)	C17—C16—C15	117.93 (14)
O3A—C36A—H36A	109.5	C21—C16—C15	122.10 (13)
O3A—C36A—H36B	109.5	C18—C17—C16	120.68 (16)
H36A—C36A—H36B	109.5	C18—C17—H17	119.7
O3A—C36A—H36C	109.5	C16—C17—H17	119.7
H36A—C36A—H36C	109.5	C19—C18—C17	119.44 (16)
H36B—C36A—H36C	109.5	C19—C18—H18	120.3
C32—O3B—C36B	118.3 (4)	C17—C18—H18	120.3
O3B—C36B—H36D	109.5	C18—C19—C20	120.84 (16)
O3B—C36B—H36E	109.5	C18—C19—H19	119.6
H36D—C36B—H36E	109.5	C20—C19—H19	119.6
O3B—C36B—H36F	109.5	C19—C20—C21	120.63 (17)
H36D—C36B—H36F	109.5	C19—C20—H20	119.7
H36E—C36B—H36F	109.5	C21—C20—H20	119.7
N1—C1—C2	101.74 (14)	C20—C21—C16	118.46 (15)
N1—C1—H1A	111.4	C20—C21—C22	121.05 (14)
C2—C1—H1A	111.4	C16—C21—C22	120.48 (12)
N1—C1—H1B	111.4	C21—C22—C24	113.74 (12)
C2—C1—H1B	111.4	C21—C22—C23	110.34 (12)
H1A—C1—H1B	109.3	C24—C22—C23	109.85 (13)
C3—C2—C1	105.74 (14)	C21—C22—H22	107.6
C3—C2—H2A	110.6	C24—C22—H22	107.6
C1—C2—H2A	110.6	C23—C22—H22	107.6
C3—C2—H2B	110.6	C22—C23—C6	113.96 (12)
C1—C2—H2B	110.6	C22—C23—H23A	108.8
H2A—C2—H2B	108.7	C6—C23—H23A	108.8
C2—C3—C4	105.04 (13)	C22—C23—H23B	108.8
C2—C3—H3A	110.7	C6—C23—H23B	108.8
C4—C3—H3A	110.7	H23A—C23—H23B	107.7
C2—C3—H3B	110.7	C25—C24—C29	118.75 (15)
C4—C3—H3B	110.7	C25—C24—C22	121.36 (14)
H3A—C3—H3B	108.8	C29—C24—C22	119.85 (16)
N1—C4—C3	104.93 (12)	C24—C25—C26	120.87 (17)
N1—C4—C5	104.42 (11)	C24—C25—H25	119.6
C3—C4—C5	113.99 (12)	C26—C25—H25	119.6
N1—C4—H4	111.0	C27—C26—C25	120.2 (2)
C3—C4—H4	111.0	C27—C26—H26	119.9
C5—C4—H4	111.0	C25—C26—H26	119.9
C30—C5—C4	115.92 (12)	C26—C27—C28	119.70 (16)
C30—C5—C6	115.96 (11)	C26—C27—Cl1	118.85 (17)
C4—C5—C6	104.90 (11)	C28—C27—Cl1	121.44 (15)
C30—C5—H5	106.4	C27—C28—C29	119.89 (17)
C4—C5—H5	106.4	C27—C28—Cl2	121.61 (14)
C6—C5—H5	106.4	C29—C28—Cl2	118.50 (17)
C23—C6—C15	107.97 (11)	C24—C29—C28	120.58 (18)
C23—C6—C5	112.44 (11)	C24—C29—H29	119.7
C15—C6—C5	109.81 (10)	C28—C29—H29	119.7
C23—C6—C7	114.45 (11)	C35—C30—C31	119.11 (15)
C15—C6—C7	110.25 (11)	C35—C30—C5	123.01 (13)
C5—C6—C7	101.81 (10)	C31—C30—C5	117.88 (13)
N1—C7—C14	115.92 (12)	C32—C31—C30	120.16 (16)
N1—C7—C8	104.01 (10)	C32—C31—H31	119.9
C14—C7—C8	100.87 (11)	C30—C31—H31	119.9
N1—C7—C6	106.94 (10)	O3B—C32—C33	125.3 (3)
C14—C7—C6	116.68 (11)	O3B—C32—C31	113.5 (3)
C8—C7—C6	111.61 (11)	C33—C32—C31	121.14 (16)
O1—C8—N2	124.93 (12)	O3B—C32—O3A	8.1 (14)
O1—C8—C7	126.47 (13)	C33—C32—O3A	117.6 (12)
N2—C8—C7	108.56 (12)	C31—C32—O3A	121.3 (11)
C10—C9—C14	122.97 (15)	C32—C33—C34	118.56 (16)
C10—C9—N2	127.28 (14)	C32—C33—H33	120.7
C14—C9—N2	109.75 (13)	C34—C33—H33	120.7
C9—C10—C11	117.24 (16)	O4—C34—C33	123.70 (16)
C9—C10—H10	121.4	O4—C34—C35	115.43 (15)
C11—C10—H10	121.4	C33—C34—C35	120.87 (16)
C12—C11—C10	121.10 (16)	C30—C35—C34	120.16 (15)
C12—C11—H11	119.4	C30—C35—H35	119.9
C10—C11—H11	119.4	C34—C35—H35	119.9
C11—C12—C13	120.54 (18)	O4—C37—H37A	109.5
C11—C12—H12	119.7	O4—C37—H37B	109.5
C13—C12—H12	119.7	H37A—C37—H37B	109.5
C14—C13—C12	119.55 (16)	O4—C37—H37C	109.5
C14—C13—H13	120.2	H37A—C37—H37C	109.5
C12—C13—H13	120.2	H37B—C37—H37C	109.5
C13—C14—C9	118.48 (14)	C7—N1—C4	106.07 (11)
C13—C14—C7	132.65 (13)	C7—N1—C1	118.19 (12)
C9—C14—C7	108.85 (12)	C4—N1—C1	105.58 (11)
O2—C15—C16	119.79 (13)	C8—N2—C9	111.80 (11)
O2—C15—C6	121.41 (12)	C8—N2—H2	124.1
C16—C15—C6	118.80 (12)	C9—N2—H2	124.1
C17—C16—C21	119.94 (14)	C34—O4—C37	117.69 (15)
			
N1—C1—C2—C3	−33.44 (19)	C16—C21—C22—C24	149.44 (15)
C1—C2—C3—C4	13.2 (2)	C20—C21—C22—C23	−155.62 (16)
C2—C3—C4—N1	12.20 (17)	C16—C21—C22—C23	25.5 (2)
C2—C3—C4—C5	−101.42 (17)	C21—C22—C23—C6	−55.47 (17)
N1—C4—C5—C30	166.65 (10)	C24—C22—C23—C6	178.36 (12)
C3—C4—C5—C30	−79.42 (16)	C15—C6—C23—C22	56.58 (15)
N1—C4—C5—C6	37.38 (13)	C5—C6—C23—C22	177.87 (12)
C3—C4—C5—C6	151.31 (13)	C7—C6—C23—C22	−66.59 (15)
C30—C5—C6—C23	−26.34 (16)	C21—C22—C24—C25	−55.9 (2)
C4—C5—C6—C23	102.91 (13)	C23—C22—C24—C25	68.31 (19)
C30—C5—C6—C15	93.90 (14)	C21—C22—C24—C29	126.54 (16)
C4—C5—C6—C15	−136.86 (12)	C23—C22—C24—C29	−109.24 (16)
C30—C5—C6—C7	−149.28 (11)	C29—C24—C25—C26	−1.2 (3)
C4—C5—C6—C7	−20.04 (13)	C22—C24—C25—C26	−178.79 (16)
C23—C6—C7—N1	−125.01 (11)	C24—C25—C26—C27	−0.6 (3)
C15—C6—C7—N1	113.06 (12)	C25—C26—C27—C28	2.2 (3)
C5—C6—C7—N1	−3.44 (13)	C25—C26—C27—Cl1	−177.52 (15)
C23—C6—C7—C14	103.36 (13)	C26—C27—C28—C29	−2.0 (3)
C15—C6—C7—C14	−18.58 (15)	Cl1—C27—C28—C29	177.76 (13)
C5—C6—C7—C14	−135.08 (11)	C26—C27—C28—Cl2	177.12 (15)
C23—C6—C7—C8	−11.87 (15)	Cl1—C27—C28—Cl2	−3.2 (2)
C15—C6—C7—C8	−133.80 (11)	C25—C24—C29—C28	1.4 (3)
C5—C6—C7—C8	109.70 (11)	C22—C24—C29—C28	179.06 (15)
N1—C7—C8—O1	55.59 (19)	C27—C28—C29—C24	0.1 (3)
C14—C7—C8—O1	176.04 (15)	Cl2—C28—C29—C24	−178.98 (13)
C6—C7—C8—O1	−59.36 (18)	C4—C5—C30—C35	−35.01 (18)
N1—C7—C8—N2	−122.56 (12)	C6—C5—C30—C35	88.67 (17)
C14—C7—C8—N2	−2.12 (15)	C4—C5—C30—C31	144.70 (14)
C6—C7—C8—N2	122.48 (12)	C6—C5—C30—C31	−91.62 (16)
C14—C9—C10—C11	2.2 (3)	C35—C30—C31—C32	0.0 (3)
N2—C9—C10—C11	−178.33 (16)	C5—C30—C31—C32	−179.76 (16)
C9—C10—C11—C12	0.9 (3)	C36B—O3B—C32—C33	−3.5 (9)
C10—C11—C12—C13	−2.7 (3)	C36B—O3B—C32—C31	177.7 (6)
C11—C12—C13—C14	1.3 (3)	C36B—O3B—C32—O3A	15 (11)
C12—C13—C14—C9	1.7 (3)	C30—C31—C32—O3B	178.6 (3)
C12—C13—C14—C7	−176.94 (18)	C30—C31—C32—C33	−0.2 (3)
C10—C9—C14—C13	−3.6 (2)	C30—C31—C32—O3A	−178.7 (17)
N2—C9—C14—C13	176.90 (15)	C36A—O3A—C32—O3B	−129 (12)
C10—C9—C14—C7	175.40 (14)	C36A—O3A—C32—C33	34 (3)
N2—C9—C14—C7	−4.13 (17)	C36A—O3A—C32—C31	−147 (2)
N1—C7—C14—C13	−65.9 (2)	O3B—C32—C33—C34	−178.1 (4)
C8—C7—C14—C13	−177.51 (18)	C31—C32—C33—C34	0.6 (3)
C6—C7—C14—C13	61.4 (2)	O3A—C32—C33—C34	179.1 (16)
N1—C7—C14—C9	115.28 (13)	C32—C33—C34—O4	178.83 (18)
C8—C7—C14—C9	3.71 (15)	C32—C33—C34—C35	−0.7 (3)
C6—C7—C14—C9	−117.37 (13)	C31—C30—C35—C34	−0.1 (2)
C23—C6—C15—O2	150.34 (15)	C5—C30—C35—C34	179.63 (14)
C5—C6—C15—O2	27.4 (2)	O4—C34—C35—C30	−179.11 (14)
C7—C6—C15—O2	−83.97 (17)	C33—C34—C35—C30	0.5 (3)
C23—C6—C15—C16	−29.28 (17)	C14—C7—N1—C4	158.89 (11)
C5—C6—C15—C16	−152.20 (13)	C8—C7—N1—C4	−91.38 (12)
C7—C6—C15—C16	96.41 (15)	C6—C7—N1—C4	26.84 (13)
O2—C15—C16—C17	4.5 (2)	C14—C7—N1—C1	40.76 (17)
C6—C15—C16—C17	−175.84 (14)	C8—C7—N1—C1	150.48 (13)
O2—C15—C16—C21	−177.35 (16)	C6—C7—N1—C1	−91.30 (15)
C6—C15—C16—C21	2.3 (2)	C3—C4—N1—C7	−160.36 (11)
C21—C16—C17—C18	1.1 (3)	C5—C4—N1—C7	−40.16 (12)
C15—C16—C17—C18	179.31 (17)	C3—C4—N1—C1	−34.16 (15)
C16—C17—C18—C19	−0.6 (3)	C5—C4—N1—C1	86.04 (13)
C17—C18—C19—C20	−0.3 (3)	C2—C1—N1—C7	160.27 (14)
C18—C19—C20—C21	0.6 (3)	C2—C1—N1—C4	41.88 (16)
C19—C20—C21—C16	−0.1 (3)	O1—C8—N2—C9	−178.40 (14)
C19—C20—C21—C22	−178.98 (18)	C7—C8—N2—C9	−0.21 (17)
C17—C16—C21—C20	−0.8 (2)	C10—C9—N2—C8	−176.74 (16)
C15—C16—C21—C20	−178.88 (16)	C14—C9—N2—C8	2.76 (18)
C17—C16—C21—C22	178.12 (15)	C33—C34—O4—C37	0.2 (3)
C15—C16—C21—C22	0.0 (2)	C35—C34—O4—C37	179.71 (17)
C20—C21—C22—C24	−31.7 (2)		

### Hydrogen-bond geometry (Å, °)

**Table:**

*D*—H···*A*	*D*—H	H···*A*	*D*···*A*	*D*—H···*A*
N2—H2···O1^i^	0.86	2.01	2.8566 (16)	166
C37—H37C···Cg1^ii^	0.93	2.75	3.493 (2)	134
C4—H4···O1	0.98	2.56	3.0989 (16)	114
C5—H5···O2	0.98	2.27	2.790 (2)	112
C13—H13···O2	0.93	2.57	3.129 (2)	119
C23—H23A···O1	0.97	2.35	3.015 (2)	125
C22—H22···N2	0.98	2.55	3.447 (2)	152

Symmetry codes: (i) −*x*+1, −*y*, −*z*+1; (ii) −*x*, −*y*+1, −*z*+2.
